# Simulation-aided infrared thermography with decomposition-based noise reduction for detecting defects in ancient polyptychs

**DOI:** 10.1186/s40494-023-01040-0

**Published:** 2023-10-20

**Authors:** Guimin Jiang, Xin Wang, Jue Hu, Yang Wang, Xin Li, Dazhi Yang, Miranda Mostacci, Stefano Sfarra, Xavier Maldague, Qiang Jiang, Hai Zhang

**Affiliations:** 1 School of Automation and Electrical Engineering, Shenyang Ligong University, Shenyang, 110159 China; 2 Centre for Composite Materials and Structures (CCMS), Harbin Institute of Technology, Harbin, 150001 China; 3 School of Information Science and Engineering, Shenyang University of Technology, Shenyang, 110870 China; 4 School of Electrical Engineering and Automation, Harbin Institute of Technology, Harbin, 150001 China; 5 Via Muranuove 64, 67043 Celano, Italy; 6 Department of Industrial and Information Engineering and Economics, University of L’Aquila, Monteluco di Roio, 67100 L’Aquila, AQ Italy; 7 Computer Vision and System Laboratory (CVSL), Department of Electrical and Computer Engineering, Laval University, Quebec City, G1V 0A6 Canada

**Keywords:** Non-destructive testing, Pulsed infrared thermography, Numerical simulation, Image processing, Tensor decomposition

## Abstract

In recent years, the conservation and protection of ancient cultural heritage have received increasing attention, and non-destructive testing (NDT), which can minimize the damage done to the test subject, plays an integral role therein. For instance, NDT through active infrared thermal imaging can be applied to ancient polyptychs, which can realize accurate detection of damage and defects existing on the surface and interior of the polyptychs. In this study, infrared thermography is used for non-invasive investigation and evaluation of two polyptych samples with different pigments and artificial defects, but both reproduced based on a painting by Pietro Lorenzetti (1280/85–1348) using the typical tempera technique of the century. It is noted that, to avoid as far as possible secondary damages done to the ancient cultural heritages, repeated damage-detection experiments are rarely carried out on the test subjects. To that end, numerical simulation is used to reveal the heat transfer properties and temperature distributions, as to perform procedural verification and reduce the number of experiments that need to be conducted on actual samples. Technique-wise, to improve the observability of the experimental results, a total variation regularized low-rank tensor decomposition algorithm is implemented to reduce the background noise and improve the contrast of the images. Furthermore, the efficacy of image processing is quantified through the structural-similarity evaluation.

## Introduction

Non-destructive testing (NDT) has become an indispensable technique in numerous fields, as it has the capability to maintain the serviceability of materials, components, and structures under test [1, 2]. Additionally, NDT techniques have been widely utilized to ensure the quality and integrity of the production process [3]. Particularly in the field of cultural heritage, of which the subjects under study have an irreplaceable and historic nature, NDT is all the more essential, as to protect the subjects from damages during the restoration and conservation process [4, 5]. Recently, a Chinese Bronze Lei was subjected using NDT techniques, enabling the detection of internal defects and ensuring the preservation of historical artifact [6, 7]. Owing to its outstanding resolution and high efficiency, as well as its ability to cover a large area in a short time period, infrared thermography (IRT), as an NDT technique, has hitherto been attracting much research interest in cultural heritage inspection [5, 8–12], in particular, it has been commonly used to evaluate the defects and damages in ancient artworks [13].

During the experiment of IRT, the temperature variation of the sample surface is recorded continuously by an infrared camera working at a fixed frequency. Defects are detected via IRT if the thermophysical properties of the defective and sound regions are different enough to produce a measurable thermal contrast. Using IRT to evaluate artworks is often perceived as more advantageous than using traditional inspection methods, owing to its non-destructive and inexpensive nature, as well as good ability to identify potential defects [14, 15]. In contrast to IRT [16–19], ultrasonic testing, which is another commonly used inspection method, requires a couplant (usually liquid) material added between the probe and the surface to be inspected. However, it is not advised to use such technique for precious/brittle test subjects, because liquid couplings can cause damages to the artwork. Another example is the penetrant testing technique, which is also problematic because of the necessity of using penetrants, which can be hard to remove.

Indeed, in restoration and preservation of artworks, the ability of detecting the defects is of primary interest, but it is also critically important to avoid secondary damages done to the artworks that are potentially caused by the NDT technique used. As such, in most cases, it is not advised to carry out repeated experiments on test subjects. The thermochromic effect constitutes a potential secondary damage while using IRT. Two ways can be used to avoid the thermochromic effect: (1) minimizing the input energy [20, 21], and/or (2) performing ad hoc numerical modeling to help obtain quantitative and reproducible results, so as to guide and optimize the procedure for testing the actual subjects [22–25]. The present work takes the second option. Numerical simulation constitutes an effective means to test and optimize the design of IRT systems [20, 24, 25], as it is not only able to mimic the experimental environment but also able to predict the experimental result. In addition, it can contribute to understanding the physical mechanism of heat transfer and radiation in the complex materials and structures, which is vital to IRT.

The test subjects of concern of this work are polyptychs, which are paintings made up of more than three panels. Polyptychs are typical anisotropic structures, since they are often constructed with multiple materials. One purpose of this work is therefore to leverage numerical simulation technique to optimize the defect detection on such anisotropic structures using IRT. The ancient polyptychs may have defects such as voids, cracks, or splitting in the interior, due to the passage of time. In the process of polyptych restoration, it is of great significance to detect different types of defects.

Two polyptych mock-ups, both based on a painting by Pietro Lorenzetti (1280/85–1348), were produced with different pigments using the typical tempera technique of the fourteenth century, and various artificial defects were introduced. Before applying IRT to the two samples, numerical simulations were conducted to reveal the physical mechanism of heat transfer and radiation. A geometric model of the samples was drawn. The computer model of the two samples was constructed in COMSOL Multiphysics®. Material properties were added to the geometrical model, and the heat transfer process was simulated to generate a temperature difference that is useful for detection.

More specifically, in order to complete the 3D modeling of the sample, the general outline of the sample was first established in the simulation environment, and then the CAD model of the detailed part of the sample was constructed. The complete sample is modeled by importing the CAD model into the simulation environment. The parameters used in the simulation process were adjusted to mimic that of the actual experimental environment.

After simulation, an infrared camera and two flash heat sources were used to establish a real experimental environment. The real temperature data of the sample surface were collected by experiments. To optimize the quality of the recorded thermal images, image denoising was carried out. The denoise technique leverages the total-variation regularized low-rank tensor decomposition, which is able to minimize Gaussian noise, impulse noise, alongside other types of noises that can potentially contaminate the images. The subsurface defects of the sample can be detected after the image processing step. Finally, the thermal images after noise reduction were further analyzed to detecting defects invisible to the naked eye. The steps mentioned above are described in more details in Sects. "Description of the samples under test and numerical simulation setup" and "Methodology."

Compared to previous infrared image detection, this study makes an outstanding contribution in denoising infrared images using Tucker decomposition. This technique is able to improve the quality and accuracy of defect detection. The novelty lies in the utilization of Tucker decomposition as a denoising method, which is advantageous in capturing and thus representing the underlying structure and spectral variations of infrared images. By utilizing the tensor-based Tucker decomposition, our method effectively reduces noise while preserving the essential information relevant to defect detection.

## Description of the samples under test and numerical simulation setup

### Realization of the samples

**Fig. 1:**
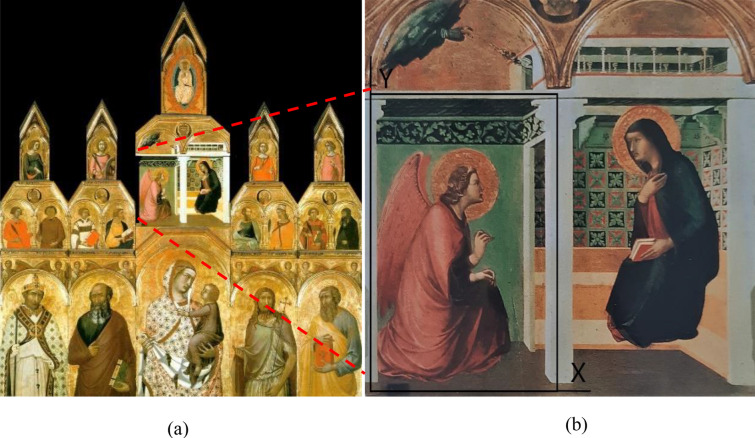
**a** A photograph of the polyptych of interest, **b** a zoomed view on the reproduced part

In order to study the detection ability of proposed method on polyptychs, two mock-ups are prepared and subjected to investigation in this study. The two mock-ups, which are both based on a fourteenth-century tempera painting, were realized by a professional restorer. The original polyptych is painted by Pietro Lorenzetti in 1320, see Fig. 1a. It is currently preserved in the Santa Maria della Pieve Church in Arezzo, Italy. The redrawn part of the painting is enlarged and shown in Fig. 1b.

The paintings were realized on supporting panels using the tempera technique that is typical in the fourteenth century. The technique uses eggs, animal glue, or vegetable glue as a binder for the pigments, and it is performed on wooden supports. To verify pros and cons of the IRT technique of interest in regard to the evaluation of defects, two similar (but not identical) painting samples were produced.

**Fig. 2:**
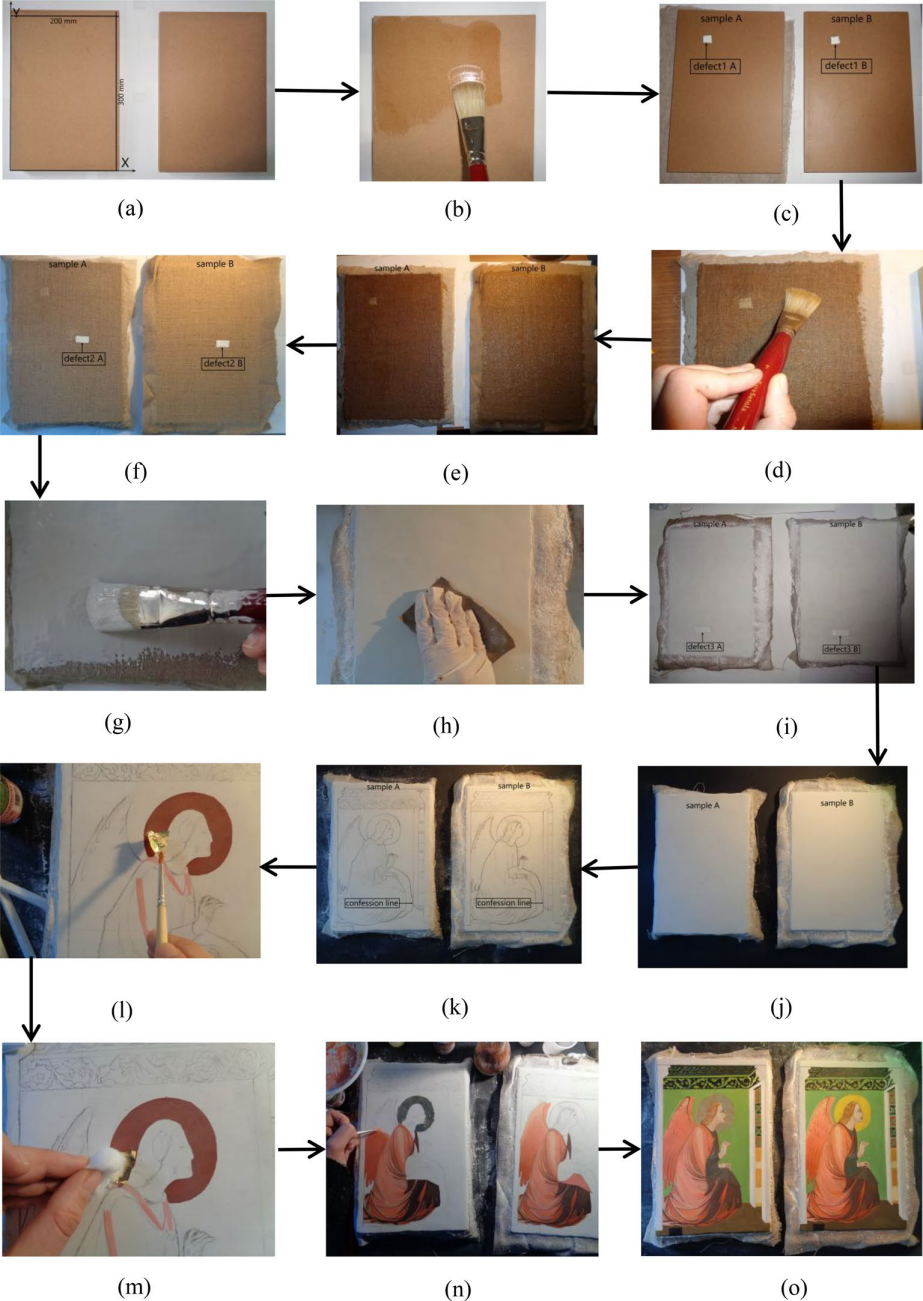
Description of the painting sample: **a** The boards are used as support, **b** applying glue with a soft brush, **c** adding the first Teflon insert (defect 1), **d** using a soft brush and applying glue on the linen canvas, **e** drying the linen canvas layer, **f** adding the second Teflon insert (defect 2), **g** applying plaster and glue, **h** sanding the plaster layer, **i** adding the third Teflon insert (defect 3), **j** sanding the second plaster layer, **k** outlying the figure, **l** the positioning of the gold leaf, **m** using a damp cotton ball to adhere the gold leaf, **n** painting different pigments for sample A and sample B, and **o** drying of the final samples. (The fabrication of the samples is completed.)

For clarity, the samples are referred to as sample A and sample B hereafter (cf. Fig. 2). Their colors were obtained using two ranges of powder pigments. For this reason, they have similar hues, but different compositions. The pigments were first diluted in water and then mixed with egg yolks, which act as adhesive. Furthermore, the preparation of plaster and glue followed the practices of the fourteenth century.

Two wooden boards, which are of the same dimensions, were used as support. The dimensions of the boards are 200 × 300 × 15 mm, see Fig. 2a. In step two, the animal glue was prepared. The rabbit glue has been selected; it was soaked in cold water for several hours by respecting a ratio of 1:7 of dry glue and water, see Fig. 2b. On the next day, the soaked glue was melted and then applied to the surface of the board with a soft brush. Finally, it was left to dry.

Defect 1 was applied on such a layer previously treated with animal glue. It simulates a splitting, because it was realized by means of a piece of twice-folded Teflon. The size of defect 1 was 11 × 15 mm. In particular, it was added between the wooden support and the canvas layer, see Fig. 2c. This defect was placed 50 mm from the *y*-axis and 236 mm from the *x*-axis, respectively, recall Fig. 2a. In sample A, defect 1 is located approximately 4 mm from the painted surface, whereas in sample B, about 5 mm.

Two types of canvas were chosen, namely, linen for sample A and flax for sample B, see Fig. 2d and e. The canvases were cut with dimensions larger than the boards, frayed along the edges, washed in hot water, dried and ironed. The thickness of the linen canvas is 1 mm, whereas the thickness of the flax canvas is 2 mm. Both the linen and the flax canvas have a regular warp-weft interweaving (1:1 ratio). In this case, the application took place with a brush having soft bristles. The layer was finally left to dry for about two days.

Once the drying of the canvas layer was completed, defect 2, namely, a second Teflon insert of 1.1 × 2.5 cm, was added, and folded once on itself, see Fig. 2f. It was placed 115 mm from the *y*-axis, 150 mm from the *x*-axis, and about 3 mm from the painting layer. The next step was the adding of the first layer of plaster. A layer of approximately 2 mm thick (Bologna plaster and rabbit glue) was applied. Gypsum was added into the glue until saturated. The application was done with a brush, see Fig. 2g. The first layer of preparation after complete drying was sanded with a fine-grained abrasive paper, see Fig. 2h. The last Teflon insert was placed on this layer, this time, without being folded back; the size of the introduced defect was 1.1 × 3.5 cm, see Fig. 2i. Defect 3 was located 65 mm from the *y*-axis and 55 mm from the *x*-axis.

Above the first layer of preparation, a second layer of plaster of Bologna mixed with rabbit glue, which has a thickness of about 1 mm, was realized. After the second layer of plaster has been evenly applied and dried, it was sanded to obtain a flat surface that is easy to paint on, see Fig. 2j. Once the procedure for preparing the support and the surface suitable for receiving the pictorial layer was completed, the restorer executed the representation of the detail by tracing the drawing with charcoal, see Fig. 2k. A *pentimento* was also mocked near the lower part of the garment.

It was decided to create the halo of the angel of sample A following the gilding technique; in contrast, yellow pigments were used for the halo in sample B. On sample A, a layer of ready-to-use red bolus (acrylic in nature) was applied by brush, on which the gold leaf was subsequently adhered, see Fig. 2l. Once the bolus had dried, it was subjected to sanding with a fine-grained abrasive paper. Small pieces to be assembled were realized with a special tool for cutting the gold leaf; they were handled with the aid of a brush and made to adhere to the bole soaked in egg white, by applying slight pressure through manually operating a cotton ball, see Fig. 2m.

For the execution of the tempera painting, egg yolk was used as a binder with the addition of two drops of vinegar, see Fig. 2n. The rendering of the figure was obtained by successive superimpositions of pictorial backgrounds with marten hair brushes, mixing each time the right amount of pigment diluted in water with the binder. The characters of sample A and sample B were painted by using different pigments. After drawing the surfaces, the fabrication of the samples was finalized, see Fig. 2o.

### Geometric modeling

**Fig. 3:**
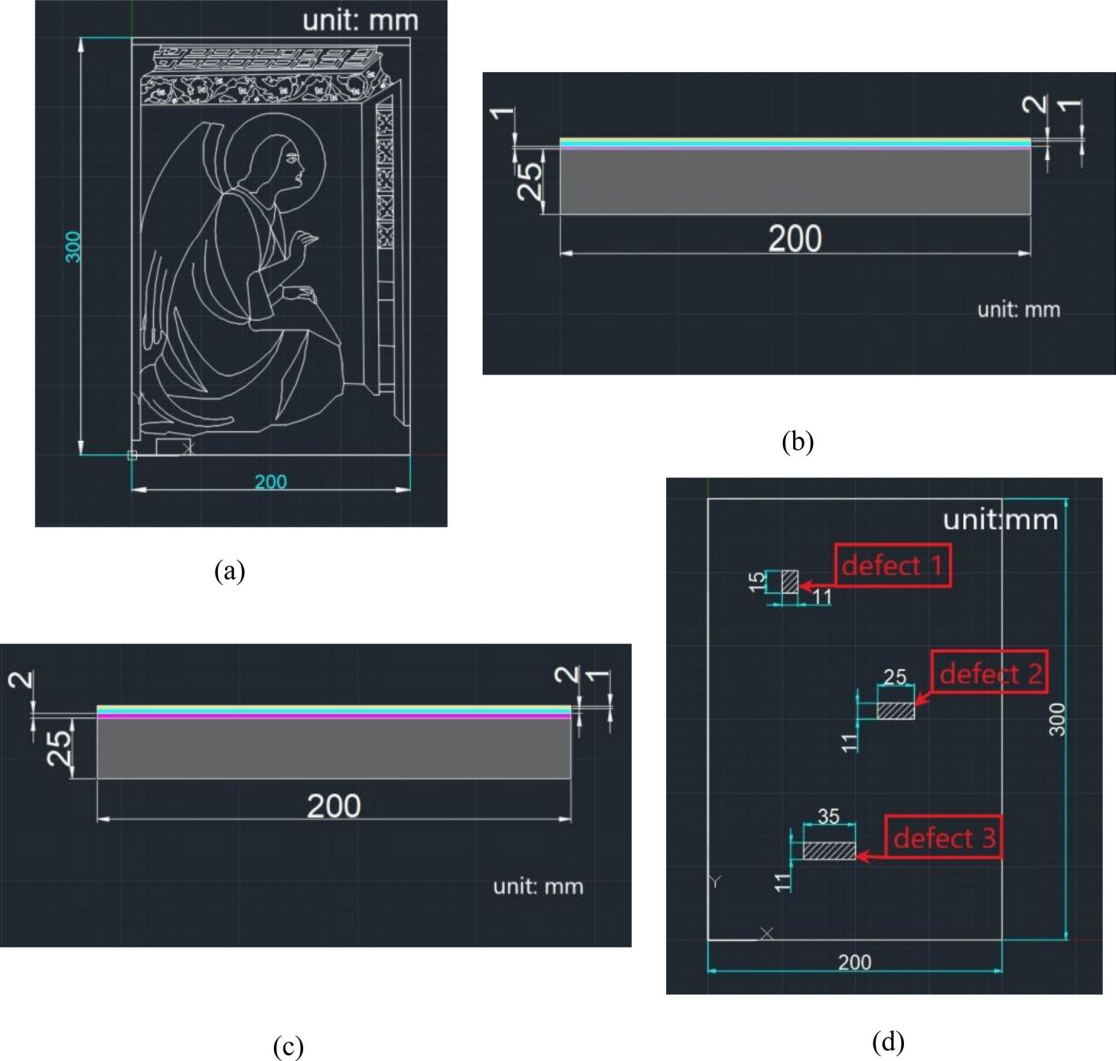
**a** The drawing of the surface of the sample, **b** side view of sample A, **c** side view of sample B, and **d** position of defects in the samples (obtained by using computer-aided designs-CAD-software). In subfigures (**b**) and (**c**), the wood panels are shown in gray, the woven fibers in magenta, and the first and second plaster preparation layers are shown in blue and yellow, respectively

This section discusses the procedure for constructing the geometric model, as to carry out numerical simulations of the temperature distribution on the surface of the samples. In this study, the geometric model was integrated with the CAD geometric design through the COMSOL Multiphysics software. During the modeling process, the defects introduced in the previous section were implemented in the COMSOL Multiphysics software. The sizes of the artificial defects are reported in Table 1. The method is based on cuboids of the sizes corresponding to the geometric dimensions of the different parts of the mock-ups. With this aim, the structure was rebuilt in an inverse manner. The positions of the cuboids in space were adjusted by changing the coordinates of the starting points. The sketch function was used to draw the outline (*x*- and *y*-axis), and then to stretch the working plane in depth (along the *z*-axis). In this way, the 3D model was built. The CAD result is shown in Fig. 3.

**Table 1:** Parameters of the geometric modeling

Position	Material	Length[mm]	Width[mm]	Thickness[mm]	Depth[mm]
(a) Specific parameters of defects in sample A					
Defect1	PTFE	11	15	0.4	4.2
Defect2	PTFE	25	11	0.2	3.2
Defect3	PTFE	35	11	0.1	1.2

(b) Specific parameters of defects in sample B					
Defect1	PTFE	11	15	0.4	5.2
Defect2	PTFE	25	11	0.2	3.2
Defect3	PTFE	35	11	0.1	1.2

Position	Sample A	Sample B
(c) Comparison between sample A and sample B		
Canvas covering	Linen canvas (thickness is 1 mm)	Flax canvas (thickness is 2 mm)
Defect 1	Depth is 4.2 mm	Depth is 5.2 mm
Defect 2	Depth is 3.2 mm	Depth is 3.2 mm
Defect 3	Depth is 1.2 mm	Depth is 1.2 mm
The angel halo	Gold	Golden ochre
Clothing	Cadmium red	Scarlet
Face	Zinc white	Zinc white
The white part of the building	Titanium white	Zinc white
The yellow part of the building	Lemon chrome	Golden ochre
The green part	Chromium hemitrioxide	Green earth
The black part	Abuser	Abuser

The material used for the samples has a great influence on both the absorption and diffusion of heat. Because the composition of mineral pigments used in fabrication process is complex, and many kinds of pigments are often mixed in the painting, this fact undoubtedly increases the difficulty of determining the specific thermal properties of materials during numerical simulation. Therefore, in the simulation process, only the main parameters of the components of the various pigments are selected to carry out the numerical simulations. The thermal parameters of the materials used in the software are summarized in Tables 2.

**Table 2:** The thermal parameters of the materials

Material	Emissivity	Density[kg/m^3]	Heat capacity[J/kg*K]	Thermal Conductivity [W/m*K]
(a) The relevant thermal parameters of pigments in sample A				
Cadmium red	0.93	4258	1490	2.7
Chromium oxide	0.92	6500	1700	2.6
Titanium white	0.91	4260	1041	0.43
Lemon chrome yellow	0.93	3895	1100	2.1
Natural ochre	0.93	5240	1010	1.16
Ivory black	0.96	2100	720	151

(b) The relevant parameters of pigments in sample B				
Scarlet	0.93	1610	920	2.4
Green Earth	0.92	2800	799	3.5
Golden ochre	0.94	4500	1010	1.16
Zinc white	0.95	5606	520	29
Ivory black	0.96	2100	720	151

## Methodology

### Simulation setting

When heat flux is applied to the surface of the sample, it follows:

1 $$-{\varvec{n}}\cdot {\varvec{q}}={q}_{0}$$

where ${\varvec{n}}$ is the angular coefficient, which represents the ratio of radiation from the surface of the heat source to the surface of the sample; ${\varvec{q}}$ is the total amount of heat generated by the heat source; ${q}_{0}$ is the power of heat flux per unit area on the surface of the sample, and the unit of ${q}_{0}$ is W/m^2^. During the first 0.02 s of the experiment, the two flashlights, which act as the heat source, provided 12,800 J. After the sample is heated, it transfers heat to the surrounding environment in the form of radiation. The value of heat given off by the sample in this radiative manner follows:

2 $${q}_{0}=\frac{E\cdot \eta \cdot \varepsilon }{t\cdot s}\cdot \alpha$$

where $E$ is the total heat generated by the flash lamps in a pulse time, $\eta$ is the thermal efficiency of the flashes, $\varepsilon$ is the emissivity of the materials, $\alpha$ is the heat loss coefficient of thermal radiation, $t$ is the duration of the flashlight heating pulse, and $s$ is the area of the sample that can absorb the radiant part of thermal energy.

In addition, a surface-to-ambient radiation component was added, to account for the energy radiated from the sample to the environment, after the heating process. The process of radiation from the sample surface to the environment follows:

3 $$-{\varvec{n}}\cdot {\varvec{q}}= \varepsilon \sigma ({T}_{\mathrm{amb}}^{4}-{T}^{4})$$

where $\varepsilon$ is the surface emissivity of the material of sample surface, of which the value depends upon the different pigments, as shown in Table 2; $\sigma$ is the Stefan–Boltzmann constant; ${T}_{\mathrm{amb}}$ is the ambient temperature; and $T$ is the temperature of the sample surface.

**Fig. 4:**
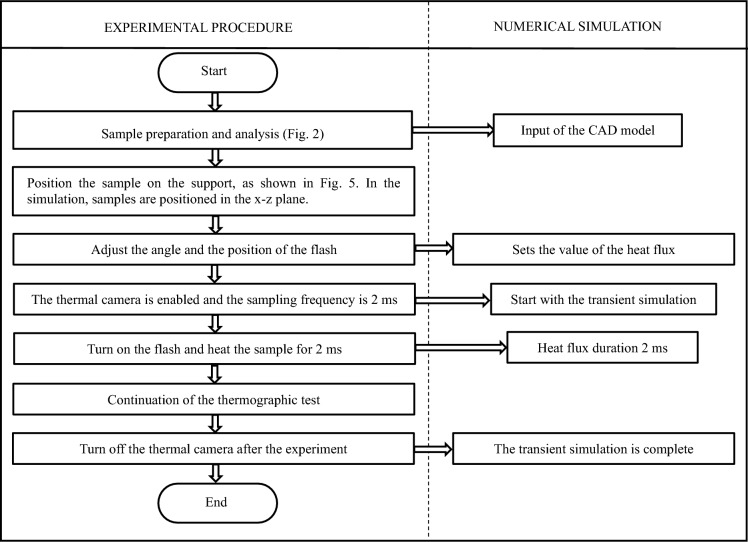
The flowchart of the IRT experiment conducted in this work

After calculation, the thermal flux value of the first study should be set to $6.0\times {10}^{5}$ W/m^2^. The function setting of heat flux in the numerical simulation is reported in Table 3. In the numerical simulation, this process was implemented as a piecewise function denoted as *P*(*t*). The flow chart of numerical simulation is shown in Fig. 4.

**Table 3:** The setting of the heat flux in the numerical simulation

Case studies	Function	Independent variable [s]	Dependent variable [W/m^2]
Setting the heat source function of sample A			
Sample A	*P*(*t*)	0–0.02	600000
		0.02–10	0

Setting the heat source function of sample B			
Sample B	*P*(t)	0–0.02	600000
		0.02–10	0

### Experimental setup

**Fig. 5:**
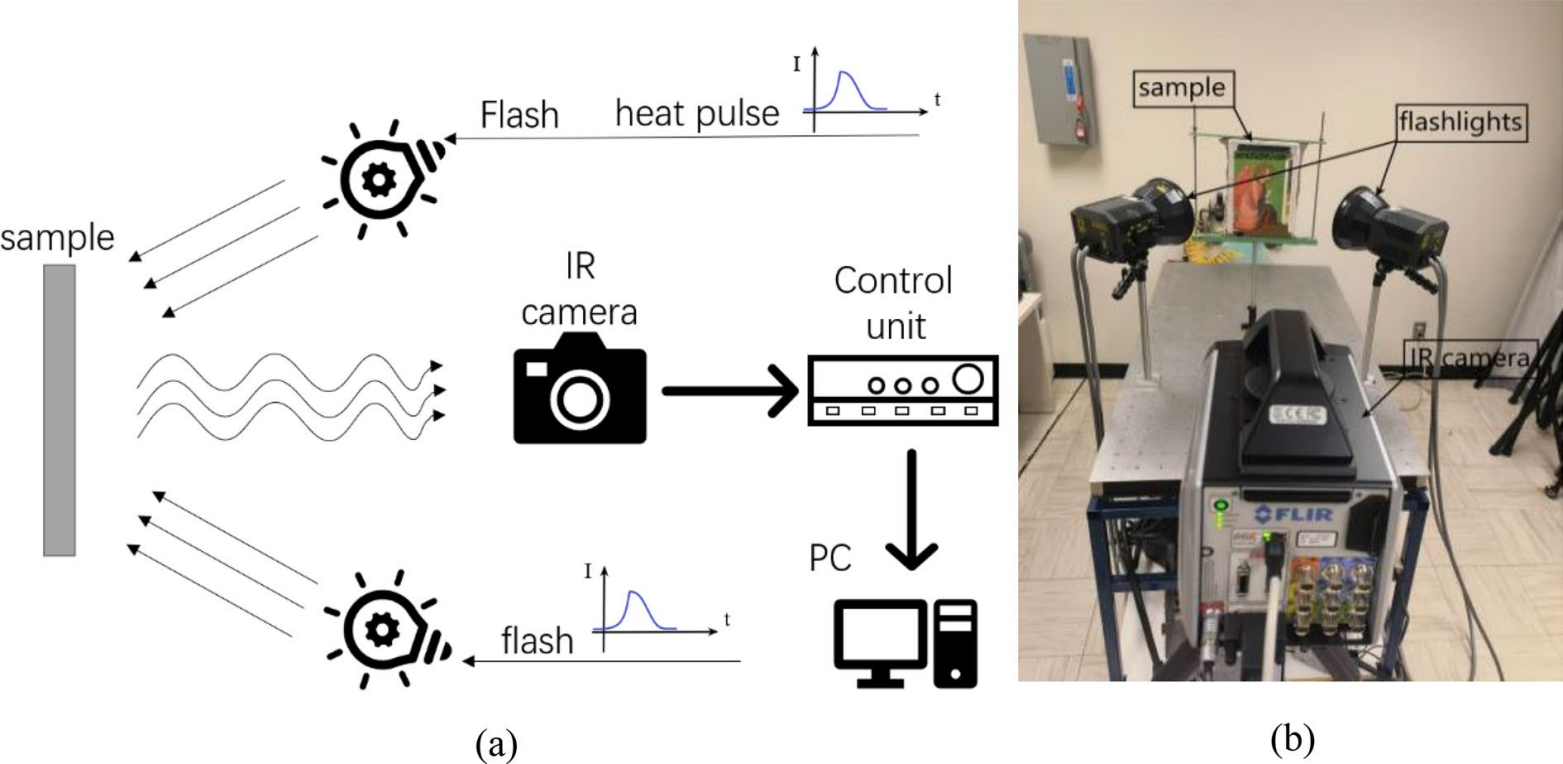
**a** The schematic configuration, and **b** the photograph of the experimental set-up

Using the active IRT approach, the thermal front can reach the sample and then diffuse inside, and thus the thermal effects due to the artificial defects could be captured by the IR detector. Each sample was heated by two flash lamps for 2 ms, each with a power of 6400 J. The diameter of the lamp holder is approximately 200 mm, whereas the position of the lamp holder was placed approximately 300 mm away from the sample surface. The schematics and the photo of the experimental setup are depicted in Fig. 5.

A mid-wave infrared camera (Flir X8501sc, 1280 × 1024 pixels, InSb detector, 3–5 µm) was used to record the temperature profile of the sample surface, and the acquisition frame rate was set to 50 Hz for 10 s. The MATLAB 2022a software was used for subsequent processing of the experimental data.

### The total-variation regularized low-rank tensor decomposition denosing system.

In [26–29], a thermographic image restoration technique based on tensor decomposition was used to reduce various types of noises contained in the experimental images. Tucker decomposition has been used in the denoising of thermographic images in [30]. In particular, it was used to describe the global correlation between each band in the non-noisy part of the thermographic image. A method of total variation regularization was used in [31, 32], whereas in [33, 34] an anisotropic spatial-spectral total variation regularization was used to represent the piecewise smoothness between the spatial domain and the spectral domain. In this study, sections of the thermographic images contain noise are subjected to ${l}_{1}$-norm regularization, as to detect sparse noise of the image. More specifically, the noise is fitted to the piecewise smoothness curve between the spatial domain and the spectral domain obtained by regularizing the spatial–spectral total variation through the noise-free region. Thus, noise can only be partially removed, which calls upon the need for further image processing. In particular, because the underlying optimization problem (see below) is non-convex, the augmented Lagrange multiplier method was used to solve the optimization.

Define a three-order tensor $y$: = {${Y}^{1},{Y}^{2},{Y}^{3},\dots , {Y}^{B}$}, where ${Y}^{i}\in {R}^{H\times W} \left(i=\mathrm{1,2},3,\dots ,B\right)$ represents the $i$^th^ frame of thermographic sequence, which was obtained by the experiment; $B$ is the number of frames; whereas $H$ and $W$ are the height and width of the image. The data obtained from the experiment can be regarded, from an image processing viewpoint, as a mixture of noiseless image and two types of noises, which may be written as:

4 $$y=X+N+S$$

where $X$ is the noiseless image, $N$ is a Gaussian noise term, and $S$ represents the sparse noise. For the detailed meaning of each symbol, the reader is referred to [31].

In order to eliminate the influence of noise on the thermographic images, a total-variation regularized low-rank tensor decomposition (LRTDTV) model is used in the noise removal process. The objective function of LRTDTV model is as follows:

5 $$\begin{gathered} \mathop {\min }\limits_{{X,N,S}} \,\,\tau \left\| X \right\|_{{{\text{SSTV}}}} {\mkern 1mu} + {\mkern 1mu} \lambda {\mkern 1mu} \left\| S \right\|_{1} {\mkern 1mu} + {\mkern 1mu} \beta \left\| N \right\|_{F}^{2} \hfill \\ {\text{s}}{\text{.}}\,{\text{t}}.\,y\, = {\mkern 1mu} \,X + N + S \hfill \\ X\, = {\mkern 1mu} \,C \times _{1} U_{1} \times _{2} U_{2} \times _{3} U_{3} \hfill \\ U_{i}^{T} \, = {\mkern 1mu} \,I{\mkern 1mu} \quad (i = {\mkern 1mu} 1,{\mkern 1mu} 2,{\mkern 1mu} 3) \hfill \\ \end{gathered}$$

where $\tau , \lambda$ and $\beta$ are the regularization parameters. The $C{\times }_{1}{U}_{1}{\times }_{2}{U}_{2}{\times }_{3}{U}_{3}$ refers to the Tucker decomposition with core tensor $C$ and factor matrices ${U}_{i}$’s of rank ${r}_{i}$’s. ${\Vert X\Vert }_{\mathrm{SSTV}}$ is the anisotropic Frobenius norm term, which takes advantages of the spatial–spectral continuity of thermographic images. The expressions of ${\Vert X\Vert }_{\mathrm{SSTV}}$ is:

6 $$\begin{aligned} \left\| X \right\|_{{{\text{SSTV}}}} = & \mathop \sum \limits_{i,j,k} \omega_{1} \left| {x_{i,j,k} - x_{i,j,k - 1} } \right| + \omega_{2} \left| {x_{i,j,k} } \right| + \\ - & x_{i,j - 1,k} \omega_{3} \left| {x_{i,j,k} - x_{i - 1,j,k} } \right| \\ \end{aligned}$$

where ${x}_{i,j,k}$ is the ${\left(i,j,k\right)}^{\mathrm{th}}$ entry of $X$; ${\omega }_{j} (j=\mathrm{1,2},3)$ is the weight along the ${j}^{\mathrm{th}}$ mode of $X$ that controls its regularization strength; and *k* represents the dimension of the thermographic image data. More specifically, this statement acknowledges that Problem (6) is a non-convex optimization problem because of the non-convex nature of Tucker decomposition. To address this, the proposal is to utilize the augmented Lagrange multiplier (ALM) method, which is a well-known optimization technique for dealing with non-convex problems. The next subsection demonstrates how ALM is able to help find a good local solution to this optimization challenge.

### Optimization procedure

By introducing some additional auxiliary variables, one can reformulate Problem (6) into an equivalent minimization problem. This reformulation could facilitate finding an alternative representation of the original optimization problem while maintaining its equivalence and providing potential benefits for optimization techniques. The reformulation is:

7 $$\begin{gathered} \mathop {\min }\limits_{{{C},{U}_{{i}} ,{X},{\mathcal{F}},S,N}} {\uptau }\left\| {\mathcal{F}} \right\|_{1} + {\uplambda }\left\| {S} \right\|_{1} + {\upbeta }\left\| {N} \right\|_{{F}}^{2} \hfill \\ \text{s.t.} \,{y} = {X} + {S} + {N},\,{X} = {\text{Z,}}\, {D}_{{\omega }} \left( {Z} \right) = {\mathcal{F}},\, \hfill \\ {X} = {C} \times_{1} {U}_{1} \times_{2} {U}_{2} \times_{3} {U}_{3} , {U}_{i}^{{\text{T}}} {U}_{i} = {I} \hfill \\ \end{gathered}$$

where ${D}_{\omega }\left(\cdot \right)=[{\omega }_{1}\times {D}_{h}\left(\cdot \right){;\omega }_{2}\times {D}_{v}\left(\cdot \right){;\omega }_{3}\times {D}_{t}\left(\cdot \right)]$ is the so-called weighted three-dimensional difference operator, and *D*_*h*_, *D*_*v*_, *D*_*t*_ are the first-order difference operators respect to three different directions. Based on the ALM methodology, Problem (8) can be transformed into minimizing the following augmented Lagrangian function:

8 $$\begin{gathered} L\left( {X,S,N,Z,{\mathcal{F}},\Gamma_{1} ,\Gamma_{2} ,\Gamma_{3} } \right) \hfill \\ = \tau \left\| {\mathcal{F}} \right\|_{1} + \lambda \left\| S \right\|_{1} + \beta \left\| N \right\|_{F}^{2} \left\langle {\Gamma_{1} ,{\text{ y}} - X - S - N} \right\rangle + \left\langle {\Gamma_{2} ,X - Z} \right\rangle \hfill \\ + \left\langle {\Gamma_{3} ,D_{\omega } \left( Z \right) - {\mathcal{F}}} \right\rangle + \frac{\mu }{2}\left( {\left\| {y - X - N} \right\|_{F}^{2} } \right. \hfill \\ \left. { + \left\| {X - Z} \right\|_{F}^{2} + \left\| {D_{\omega } \left( Z \right) - {\mathcal{F}}} \right\|_{F}^{2} } \right) \hfill \\ \end{gathered}$$

Under the constraints $X=C{\times }_{1}{U}_{1}{\times }_{2}{U}_{2}{\times }_{3}{U}_{3}$,and ${{U}_{i}}^{T}{U}_{i}=I$, where $\mu$ is the penalty parameter, and ${\Gamma }_{i} (i=\mathrm{1,2},3)$ are the Lagrange multipliers. Therefore, during the optimization process, one can employ an alternative approach to optimize the augmented Lagrangian function (9) by updating one variable at a time while keeping the others fixed. In the iteration, the variables related to Problem (6) can be updated using the procedure outlined below. This iterative process could efficiently solve the optimization problem by updating variables in a stepwise manner while considering the constraints introduced by ALM.

1. Update $C$*, *${U}_{i}$*,* $X$: Extracting all terms containing $X$ from the augmented Lagrangian function (9), one needs to solve:

9 $$\begin{gathered} \mathop {\text{min}}\limits_{{\begin{array}{*{20}c} {U_{i}^{T} U_{i} = I} \\ {X = C \times_{1} U_{1} \times_{2} U_{2} \times_{3} U_{3} } \\ \end{array} }} \left\langle {\Gamma_{1}^{\left( k \right)} ,y - X - S^{\left( k \right)} - N^{\left( k \right)} } \right\rangle \hfill \\ + \,\left\langle {\Gamma_{2}^{\left( k \right)} ,X - Z^{\left( k \right)} } \right\rangle \hfill \\ + \frac{\mu }{2}\left( {\left\| {y - X - S^{\left( k \right)} - N^{\left( k \right)} } \right\|_{F}^{2} + \left\| {X - Z^{\left( k \right)} } \right\|_{F}^{2} } \right) \hfill \\ \end{gathered}$$

This problem can be readily converted into the following equivalent formulation:

10 $$\begin{gathered} \mathop {\min }\limits_{{U_{i}^{T} U_{i} }} { }\left\| {\mu C \times_{1} U_{1} \times_{2} U_{2} \times_{3} U_{3} - \frac{1}{2}\left( {y - S^{\left( k \right)} } \right.} \right. \hfill \\ \left. {\left. { - N^{\left( k \right)} + Z^{\left( k \right)} + \left( {\Gamma_{1}^{\left( k \right)} - \Gamma_{2}^{\left( k \right)} } \right)/\mu } \right)} \right\|_{F}^{2} \hfill \\ \end{gathered}$$

By using the classic higher-order orthogonal iteration algorithm, ${C}^{(k+1)}$ and ${U}_{i}^{(k+1)} (i=\mathrm{1,2},3)$ can be easily obtained, such that $X$ can be updated as follows:

11 $${X}^{\left(k+1\right)}={C}^{\left(k+1\right)}{\times }_{1}{U}_{1}^{\left(k+1\right)}{\times }_{2}{U}_{2}^{\left(k+1\right)}{\times }_{3}{U}_{3}^{\left(k+1\right)}$$

(2) Update $Z$: By extracting all the terms containing $Z$ from the augmented Lagrangian function (9), one can derive:

12 $$\begin{aligned} Z^{k + 1} = & \mathop {\text{argmin}}\limits_{Z} \left\langle {\Gamma_{2}^{\left( k \right)} ,X^{{\left( {k + 1} \right)}} - Z} \right\rangle \\ + & \left\langle {\Gamma_{3}^{\left( k \right)} ,D_{\omega } \left( Z \right) - {\mathcal{F}}^{\left( k \right)} } \right\rangle \\ + & \frac{\mu }{2}\left( {\left\| {X^{{\left( {k + 1} \right)}} - Z} \right\|_{F}^{2} + \left\| {D_{\omega } \left( Z \right) - {\mathcal{F}}} \right\|_{F}^{2} } \right) \\ \end{aligned}$$

The optimization of this problem can be translated as solving the linear system:

13 $$\begin{aligned} \left( {\mu I + \mu D_\omega ^*{D_\omega }} \right)Z =\, & \mu {X^{\left( {k + 1} \right)}} \\ + \, & \mu D_\omega ^*\left( {{\mathcal{F}^{\left( k \right)}}} \right) \\ + & \,\Gamma _2^{\left( k \right)} - D_\omega ^*\left( {\Gamma _3^{\left( k \right)}} \right) \\ \end{aligned}$$

where ${\mathrm{D}}_{\upomega }^{*}$ represents the adjoint operator of ${D}_{\omega }$. Since the block cyclic structure of the matrix corresponding to the operator ${D}_{\omega }^{*}{D}_{\omega }$, it can be diagonalized with a three-dimensional matrix. Therefore, can be deduced:

14 $$\left\{\begin{array}{c}H_Z=\mu {X}^{\left(k+1\right)}+\mu {X}_{\omega }^{*}\left({\mathcal{F}}^{\left(k\right)}\right)+{\Gamma }_{2}^{\left(k\right)}-{D}_{\omega }^{*}\left({\Gamma }_{3}^{\left(k\right)}\right)\\ {T}_{Z}={\omega }_{1}^{2}{\left|\mathrm{fftn}\left({D}_{h}\right)\right|}^{2}+{\omega }_{2}^{2}{\left|\mathrm{fftn}\left({D}_{v}\right)\right|}^{2}+{\omega }_{3}^{2}{\left|\mathrm{fftn}\left({D}_{t}\right)\right|}^{2} \\ {Z}^{\left(\mathrm{k}+1\right)}=\mathrm{ifftn}\left(\frac{\mathrm{fftn}\left({H}_Z\right)}{{\mu }_{1}+{\mu }_{{T}_{Z}}}\right)\end{array}\right.$$

where fftn and ifftn represent fast three-dimensional Fourier transform and its inverse transform respectively, ${\left|\cdot \right|}^{2}$ is the element-wise square, and the division is based on element- wise.

(3) Update $\mathcal{F}$: Extracting all terms containing $\mathcal{F}$ from function (9), one can get:

15 $$\begin{aligned} {\mathcal{F}}^{{\left( {k + 1} \right)}} = & \mathop {\text{argmin}}\limits_{{\mathcal{F}}} { }\tau \left\| {\mathcal{F}} \right\|_{1} \\ + & \left\langle {\Gamma_{3}^{\left( k \right)} ,D_{\omega } \left( {Z^{{\left( {k + 1} \right)}} } \right) - {\mathcal{F}}} \right\rangle \, + \,\frac{\mu }{2}\left\| {D_{\omega } \left( {Z^{{\left( {k + 1} \right)}} } \right) - {\mathcal{F}}} \right\|_{F}^{2} \\ = & \mathop {\text{argmin} }\limits_{{\mathcal{F}}} \tau \left\| {\mathcal{F}} \right\|_{1} + \frac{\mu }{2}\left\| {{\mathcal{F}} - \left( {D_{\omega } \left( {Z^{{\left( {k + 1} \right)}} } \right) + \frac{{M_{3}^{\left( k \right)} }}{\mu }} \right)} \right\|_{F}^{2} \\ \end{aligned}$$

By incorporating the soft-thresholding operator, a widely used mathematical tool in signal processing and optimization, one can address the non-convex nature of the problem. This operator aids in controlling the regularization strength of the optimization process and facilitates the derivation of more desirable local solutions.

16 $${\text{R}}_{\Delta } \left( {\text{x}} \right)\left\{ {\begin{array}{*{20}c} {x - \Delta {\mkern 1mu} \quad } & {if{\mkern 1mu} x > \Delta } \\ {x + \Delta \quad {\mkern 1mu} } & {if{\mkern 1mu} x\, < \,\Delta } \\ {0{\mkern 1mu} } & \text{otherwise} \\ \end{array} } \right.$$

where $x\in R$ and $\Delta >0$, then ${\mathcal{F}}^{\left(k+1\right)}$ can be updated as:

17 $${\mathcal{F}}^{k+1}={R}_{\frac{\tau }{\mu }}\left({D}_{\omega }\left({Z}^{\left(k+1\right)}\right)+\frac{{\Gamma }_{3}^{\left(k\right)}}{\mu }\right)$$

(4) Update $S$: Similarly, one may consider:

18 $$\begin{aligned} {S^{\left( {k + 1} \right)}} = & \mathop {\text{argmin}}\limits_S \lambda {\left\| S \right\|_1} + \left\langle {\Gamma _1^{\left( k \right)},y - {X^{\left( {k + 1} \right)}} - S - {N^{\left( k \right)}}} \right\rangle \\ + & \frac{\mu }{2}\left\| {y - {X^{\left( {k + 1} \right)}} - S - {N^{\left( k \right)}}} \right\|_F^2 \\ = & \mathop {\text{argmin}}\limits_S \lambda {\left\| S \right\|_1} \\ + & \frac{\mu }{2}\left\| {S - \left( {y - {X^{\left( {k + 1} \right)}} - {N^{\left( k \right)}} + \frac{{\Gamma _1^{\left( k \right)}}}{\mu }} \right)} \right\|_F^2 \\ \end{aligned}$$

By leveraging the previously introduced soft-thresholding operator, the solution to the above problem can be expressed in a more tractable and efficient manner, which is:

19 $$S^{(k+1)} \, = \,R_{{\frac{\lambda }{\mu }}} \left( {y - X^{{(k + 1)}} - N^{(k)} + \frac{{{\text{M}}_{1}^{(k)} }}{\mu }} \right)$$

(5) Update $N$: By isolating the terms involving variable $N$ in the augmented Lagrangian function (9), one obtains a more concise and focused representation, allowing for a more efficient and targeted optimization approach for handling $N$, indicated as:

20 $$\begin{aligned} N^{(k + 1)} \, = & \,\mathop {\text{argmin}}\limits_{N} \,\beta \left\| N \right\|_{F}^{2} \\ + & \,\,\left\langle {\Gamma_{1}^{\left( k \right)} ,y - X^{{\left( {k + 1} \right)}} - S^{{\left( {k + 1} \right)}} - N} \right\rangle \\ \, + & \,\frac{\mu }{2}\left\| {y - X^{{\left( {k + 1} \right)}} - S^{{\left( {k + 1} \right)}} - N} \right\|_{F}^{2} \\ = \, & \mathop {\text{argmin}}\limits_{N} \,\left( {\beta + \frac{\mu }{2}} \right)\left\| {N - \frac{{\mu \left( {y - X^{{\left( {k + 1} \right)}} - S^{{\left( {k + 1} \right)}} } \right) + \Gamma_{1}^{\left( k \right)} }}{\mu + 2\beta }} \right\|_{F}^{2} \\ \end{aligned}$$

By performing straightforward calculations, the solution for the variable can be obtained as follows:

21 $${N}^{k+1}=\frac{\mu \left(y-{X}^{\left(k+1\right)}-{S}^{\left(k+1\right)}\right)+{M}_{1}^{\left(k\right)}}{\mu +2\beta }$$

(6) Updating the multipliers: In the ALM method, the multipliers are updated iteratively using specific equations, which are part of the optimization process to solve the given problem, be expressed as follows:
  22 $$\left\{\begin{array}{c}{\Gamma }_{1}^{\left(k+1\right)}={\Gamma }_{1}^{\left(k\right)}+\mu \left(y-X^{\left(k+1\right)}-S^{\left(k+1\right)}-N^{\left(k+1\right)}\right)\\ {\Gamma }_{2}^{\left(k+1\right)}={\Gamma }_{2}^{\left(k\right)}+\mu \left(X^{\left(k+1\right)}-Z^{\left(k+1\right)}\right)\\ {\Gamma }_{3}^{\left(k+1\right)}={\Gamma }_{3}^{\left(k\right)}+\mu \left({D}_{\omega }\left({Z}^{\left(k+1\right)}\right)-{\mathcal{F}}^{\left(k+1\right)}\right)\end{array}\right.$$

In summary, an ALM-based method has been developed to solve the proposed LRTDTV model, cf. Problem (6); this procedure is outlined in Algorithm 1. The solver takes in as inputs the noisy image $y\in {R}^{M\times N\times p}$, desired rank $[{r}_{1},{r}_{2},{r}_{3}]$ for Tucker decomposition, the stopping criteria, and the regularized parameters $\tau$, $\lambda$ and $\beta$. Due to the inherent proportional relationship among these three parameters, one can simply set $\tau =1$ and then tune $\lambda$ and $\beta$. For another important parameter $u$, it is first initialized as $u=1{0}^{-2}$ and then updated via $u=\mathrm{min}(\rho u,{u}_{\mathrm{max}})$ in each iteration. The approach of adaptively determining the variable $u$ has been commonly employed in ALM-based methods, effectively promoting the convergence of the algorithm.

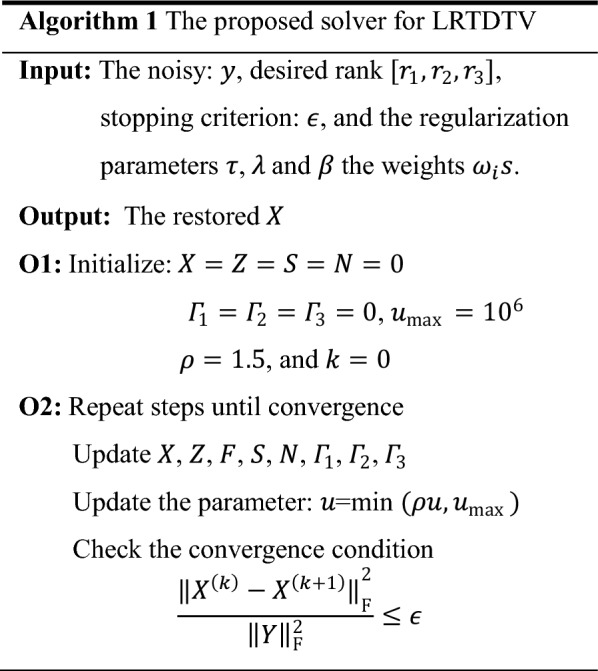



By capturing the spatial and spectral information of the thermographic images, this method is able to eliminate the noise contained in the images. Firstly, Tucker decomposition of thermographic images was carried out by using the continuity of all pixels in the spectral domain and the correlation between the spatial domain and spectral domain. The ${l}_{1}$ regularization has been used to detect the noise term. If the noise has been detected by the ${l}_{1}$ regularization system, the above-mentioned spatial–spectral total variation regularization system is used to characterize the piecewise smooth structure between the spatial domain and the spectral domain, so as to help remove Gaussian noise mixed in the image. In addition, some heavy Gaussian noises were further removed by the Frobenius norm term.

After the LRTDTV denoise processing, a Fourier transform was performed to further improve the contrast and clarity of the image. The specific details of the algorithm are more described in the study of Wang Y [34], to whom the readers are referred.

## Result and discussion

In this section, the results are displayed, alongside thorough analyses on the efficacy of the proposed method. Two types of analyses are made, namely, visual judgment and quantitative assessment, which are depicted in the following two subsections.

### Visual judgment

**Fig. 6:**
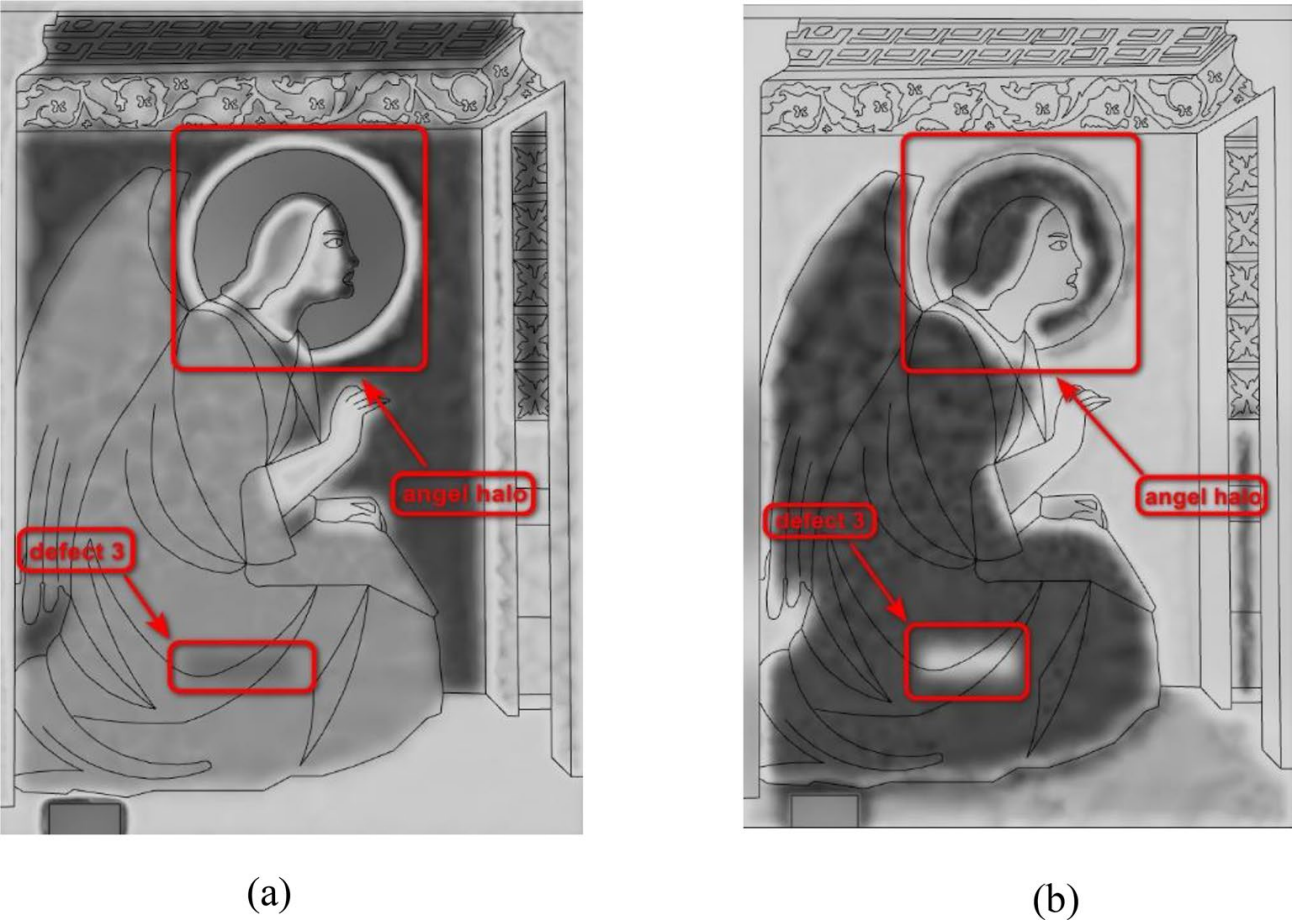
Simulation results for: **a** sample A and **b** sample B

The results of numerical simulations are shown in Fig. 6. For sample A, a large portion of IR waves was reflected during the heating process due to the low emissivity value of gold leaf, making it difficult to detect the structure beneath. On the contrary, because the halo of sample B was painted with mineral pigments with high emissivity value, this phenomenon does not occur.

In Fig. 6, defects 3 can be clearly observed. In the simulation models, defect 3 is located 65 mm from the *y*-axis and 55 mm from the *x*-axis. The location of defect 3 detected by numerical simulation is consistent with that introduced in the actual sample.

**Fig. 7:**
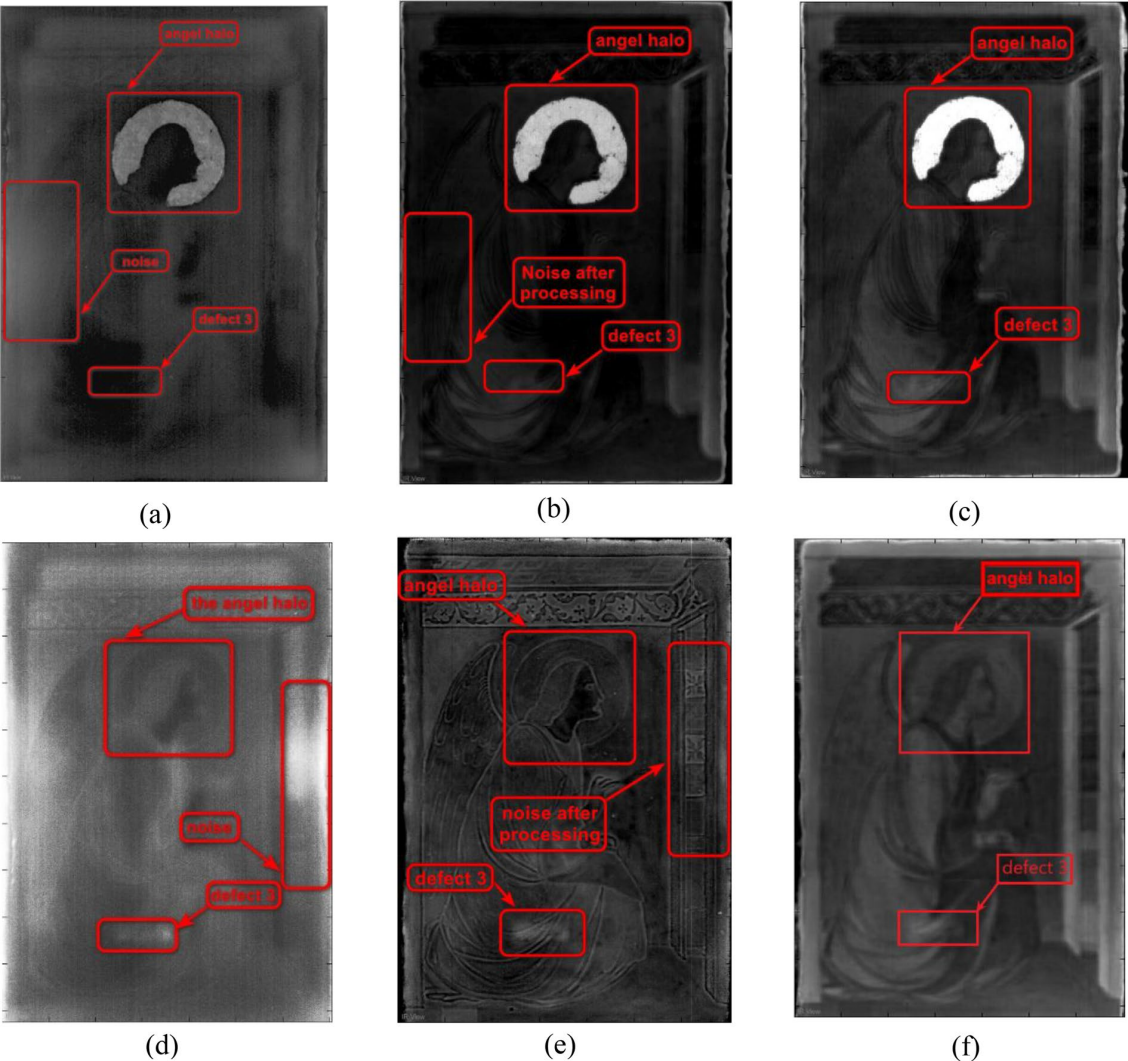
IRT experimental results: **a** the raw image of sample A, **b** the image of sample A after Fourier transform, **c** the image of sample A after LRTDTV de-noise and Fourier transform, **d** the raw image of sample B, **e** the image of sample B after Fourier transform, and **f** the image sample B after LRTDTV de-noise and Fourier transform

Figure 7 shows the thermal images collected by the infrared camera, and processed using different algorithms. Figure 7a and d correspond to the raw images of the two samples, whereas Fig. 7c and f are the final images obtained by applying LRTDTV noise reduction and Fourier transform. To benchmark the proposed method, Fig. 7b and e show the images that undergo a Fourier transform, but without applying the LRTDTV model.

By directly eyeballing the raw images shown in Fig. 7a and d, it is difficult to notice any defect. Figure 7b and e, on the other hand, show the images processed by Fourier transform. It can be found that most of the Gaussian noise in the images can be removed, and the position of defect 3 can be seen vaguely. As such, performing an LRTDTV denoising on the raw images followed by a Fourier transform is thought beneficial. The images processed by the LRTDTV model and Fourier transform, which leads to further improvements in the contrast and sharpness of the images, are shown in Fig. 7c and f.

It can be seen from Fig. 7c that the temperature of the angel halo contrasts that of the sound area, which is due to the presence of gold foil, and the maximum temperature difference reaches 0.8°C. However, this phenomenon does not exist on the surface of sample B, see Fig. 7f, which is consistent with the results obtained by numerical simulation. Similarly, the positions of defect 3 in Fig. 7c and f are consistent with the predicted ones shown in Fig. 6a and b.

### Quantitative assessment

In this work, quantitative assessments are performed for two purposes, one is to check whether the simulation results are consistent with the experimental outcome, and the other is to check whether the proposed method is able to enhance the defect detection.

#### Comparison between the simulated and experimental data

For an objective assessment of the similarity between simulated and experimental images, a total of three metrics, namely, structural similarity (SSIM), peak signal to noise ratio (PSNR), and *Erreur Relative Globale Adimensionnelle de Synthèse* (ERGAS), which are able to quantify the degree of similarity between the two image sets, are employed. Their computation methods are detailed below:

SSIM, which is an index to measure the similarity of two pictures, is computed as:

23 $$\mathrm{SSIM}=\frac{(2{\mu }_{x}{\mu }_{y}+{C}_{1})(2{\sigma }_{xy}+{C}_{2})}{({\mu }_{x}^{2}{+\mu }_{y}^{2}+{C}_{1})({\sigma }_{x}^{2}+{\sigma }_{y}^{2}+{C}_{2})}$$

where ${\mu }_{x}$ and ${\mu }_{y}$ are the brightness values along the horizontal and vertical directions of the gray-level average image, respectively; ${\sigma }_{x}$ and ${\sigma }_{y}$ are the standard deviations of the horizontal and vertical directions of the gray-level average image, respectively, representing the contrast of the image; and ${C}_{1},{C}_{2},{C}_{3}$ are positive constants [35].

PSNR, which a full-reference image quality evaluation index, is calculated as follows

24 $$\mathrm{PSNR}=10{\mathrm{log}}_{10}\,\left(\frac{{2}^{n}-1}{\mathrm{MSE}}\right)$$

where MSE stands for “mean square error,” which is given as:

25 $$\mathrm{MSE}=\frac{1}{H\times W}\sum_{i=1}^{H}\sum_{j=1}^{W}{\left[X\left(i,j\right)-Y\left(i,j\right)\right]}^{2}$$

where $H$ and $W$ are the height and width of the image respectively; $n$ is the number of bits per pixel, which is generally 8, which means that the pixel gray level is 256. The unit of PSNR is dB, the larger the value, the smaller the distortion.

ERGAS, which stands for “square root of average relative global error,” is another indicator used to assess image quality. This measure is computed as:

26 $$\mathrm{ERGAS}=\left(\frac{1}{p}\right)\times \sqrt{\sum_{i=1}^{p}\frac{{\mathrm{RMSE}\left(i\right)}^{2}}{{\mu \left(i\right)}^{2}}}$$

where $p$ is the number of bands, $\mathrm{RMSE}\left(i\right)$ is the root-mean-square error of the ${i}^\text{th}$ band, and $\mu \left(i\right)$ is the mean of the ${i}^\text{th}$ band. The smaller the value of the ERGAS value is, the better the image quality is said to be.

The raw images and the images after LRTDTV denoising and Fourier transform are used as *X* and *Y*, respectively in Eqs. (24)–(27). The quantitative assessment values are shown in Table 4.

**Table 4:** The quantitative assessment values for the simulated and experimental images

	SSIM	PSNR	ERGAS
Simulation for sample_A	0.95	49.91	26.31
Experiment for sample_A	0.88	46.19	29.24
Simulation for sample_B	0.83	22.02	25.01
Experiment for sample_B	0.78	22.02	25.01

**Fig. 8:**
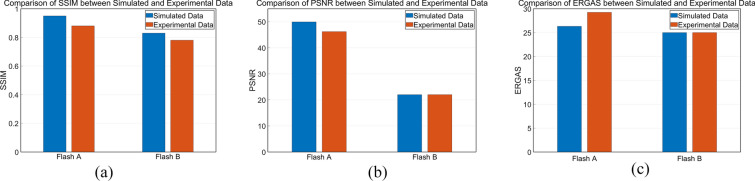
Comparison of simulation and experimental data using: **a** SSIM, **b** PSNR, and **c** ERGAS

In Fig. 8, it can be concluded that the simulated and experimental data show similar trends. The quantitative comparison can confirm the simulations as a reliable guide to the experiments.

#### Quantitative assessment of the efficacy of the proposed method

Table 5 shows the SSIM values of defect 3. It can be concluded that the SSIM values are significantly reduced applying LRTDTV denoising and Fourier transform, which indicates that the contrast of defect 3 is enhanced correspondingly.

**Table 5:** The SSIM between defect 3 and its sound area, before and after applying the proposed processing method

Position	Raw image of sample A	Processed image of sample A	Raw image of sample B	Processed image of sample B
Defect 3	0.65	0.64	0.66	0.35

For the purpose of comparison, Michelson contrast (MC), histogram flatness measure (HFM) and histogram spread (HS) are also used in this work. These metrics are used to evaluate the contrast between the defective regions of the image and the sound area.

MC is defined as:

27 $$\mathrm{MC}=\frac{{\mathrm{I}}_{\mathrm{max}}-{\mathrm{I}}_{\mathrm{min}}}{{\mathrm{I}}_{\mathrm{max}}+{\mathrm{I}}_{\mathrm{min}}}$$

where ${\mathrm{I}}_{\mathrm{max}}$ and ${\mathrm{I}}_{\mathrm{min}}$ are the largest and smallest pixel values of the selected area in the image respectively. $\mathrm{I}$ denotes the pixel value of the selected area. The range of the MC is [0,1]. More specifically, when the gray scales of the brightest and darkest pixels of an image are both 128, the image has no contrast, i.e., $\mathrm{MC}$=0. When the gray scale of the brightest pixel is 255 and that of the darkest pixel is 0, the image contrast is the highest, i.e., $\mathrm{MC}$=1.

HFM is defined as the ratio between the geometric and the arithmetic means of the histogram values [36], denoted as $h\left(x\right)$. It can provide the insights into the image's overall contrast and tonal distribution characteristics. It aids in assessing the degree of balance in pixel intensity representation, contributing to improved image analysis and interpretation. It is defined as:

28 $$\begin{aligned} {\text{HFM}} = & \frac{{G.M.{\text{ of histogram count}}}}{{A.M.{\text{ of histogram count}}}} \\ = & \frac{{\left( {\mathop \prod \nolimits_{i = 1}^{n} x_{i} } \right)^{\frac{1}{n}} }}{{\frac{1}{n}\mathop \sum \nolimits_{i = 1}^{n} x_{i} }} \\ \end{aligned}$$

where ${x}_{i}$ represents the count of pixel intensities in the *i*th histogram partition, $i$ represents the ${i}^{\text{th}}$ histogram partition, and $n$ represents the total number of histogram partitions.

As a basic feature, the geometric mean of a dataset is always less than or equal to its arithmetic mean, resulting in HFM values in the range [0, 1]. A higher HFM value indicates a more uniform intensity distribution across the image, while a lower HFM value indicates a less uniform pixel intensity distribution.

HS is a valuable measure for analyzing the spread and contrast characteristics of digital images based on their histogram characteristics. It can be used to distinguish images with different contrast and intensity distributions. It is calculated as the ratio of the interquartile range to the histogram range. HS can be defined as:

29 $$\begin{aligned} {\text{HS}} = \,& \frac{{\text{Quartile range of histogram}}}{{\text{Range of pixel values}}} \\ =\, & \frac{{\left( {3^{{{\text{rd}}}} {\text{ quartile}} - 1^{{{\text{st}}}} {\text{ quartile}}} \right){\text{ of histogram}}}}{{\left( {{\text{max}} - {\text{min}}} \right){\text{ of the pixel value range}}}} \\ \end{aligned}$$

where the $\mathrm{Quartile range}$ in the numerator represents the difference between the ${3}^{\mathrm{rd}}$ quartile (corresponding to the histogram partition where 75% of the cumulative histogram maximum is located) and the ${1}^{\mathrm{st}}$ quartile (corresponding to the histogram partition where 25% of the cumulative histogram maximum is located). The range in the denominator is the difference between the maximum and minimum intensity possible for the image (e.g., for an 8-bit image, the minimum intensity is 0 and the maximum intensity is 255). For images with multimodal histograms, the HS value is in the range (0, 1). The HS value gives an idea about the contrast characteristics of the image. Images with low contrast, with narrow histograms and high peaks, tend to have low HS values, while images with high contrast, with wide and flat histograms, have high HS values.

**Fig. 9:**
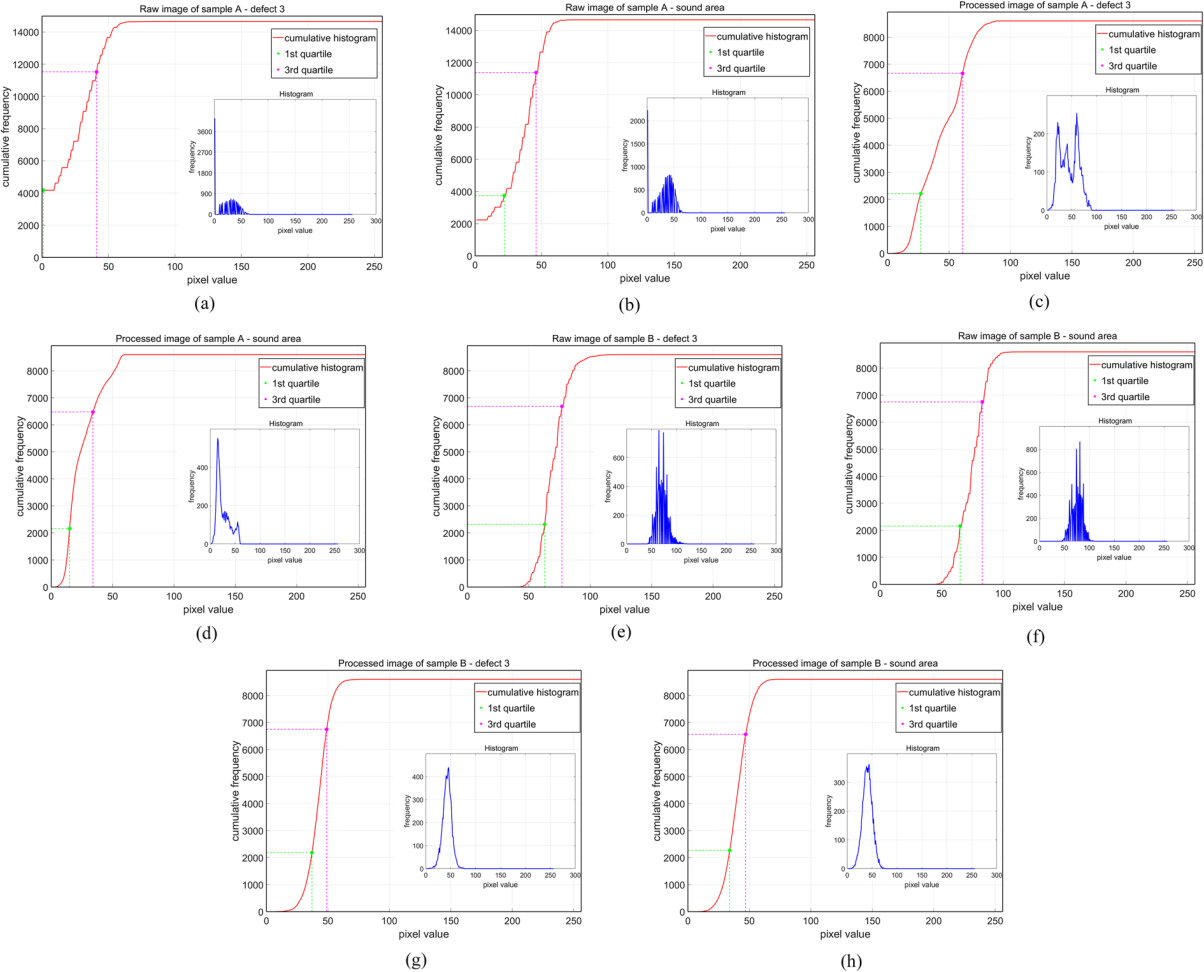
Cumulative histogram curves for: **a** defect 3 in the raw image of sample A, **b** sound area in the raw image of sample A, **c** defect 3 in the image of sample A after applying LRTDTV denoising and Fourier transform, **d** sound area in the raw image of sample A after applying LRTDTV denoising and Fourier transform, **e** defect 3 in the raw image of sample B, **f** sound area in the raw image of sample B, **g** defect 3 in the image of sample B after applying LRTDTV denoising and Fourier transform, **h** sound area in the raw image of sample B after applying LRTDTV denoising and Fourier transform

The histogram counts, quartiles, and pixel ranges are used to calculate the difference between the ${3}^{\mathrm{rd}}$ quartile and the 1st quartile in Fig. 7. The computational results are shown in Fig. 9:

Table 6 shows the computational values. After applying LRTDTV denoising and Fourier transform for sample A, the MC values decrease and the HFM and HS values increase. This indicates that the image of sample A becomes blurred, but the range of pixel intensity is expanded and more uniform. The values of MC, HFM and HS for the difference between defect 3 and sound area increase, which makes defect 3 more clearly detected in sample A.

**Table 6:** Computational values of defect 3

	Defect 3	sound area
MC of raw image of sample A:	0.45	0.46
MC of processed image of sample A:	0.27	0.34
HFM of raw image of sample A:	3.76 $\times$ 10^–7^	2.51 $\times$ 10^–7^
HFM of processed image of sample A:	2.96 $\times$ 10^–6^	1.18 $\times$ 10^–6^
HS of raw image of sample A:	0.06	0.08
HS of processed image of sample A:	0.09	0.04
MC of raw image of sample B:	0.08	0.05
MC of processed image of sample B:	0.23	0.21
HFM of raw image of sample B:	4.98 $\times$ 10^–7^	5.31 $\times$ 10^–7^
HFM of processed image of sample B:	1.84 $\times$ 10^–6^	3.97 $\times$ 10^–6^
HS of raw image of sample B:	0.06	0.05
HS of processed image of sample B:	0.04	0.06

For sample B after processing, the HS values decrease and the HFM and MC values increase. This indicates that the image becomes clearer after applying LRTDTV denoising and Fourier transform, but the range of pixel intensity is reduced. The values of MC, HFM, and HS for the difference between defect 3 and sound area increase, which leads to a more uneven distribution of defect 3 in the histogram Therefore, it is easier to be detected in Sample B.

These findings indicate that the proposed processing method enhances the image contrast, and thus improve the capability of defect detection. On the other hand, it also leads to more uneven distribution of pixel intensity. These make some specific features more visual, and thus more objective.

In short, the proposed method, which combines numerical simulation, infrared thermal imaging, and image processing, can accurately detect defects located at both the surface and interior of ancient artworks, while protecting them to the greatest extent possible. Because of the steps of image processing, the clarity of thermal images can be improved, so that those damages and defects that are otherwise not easy noticeable can be detected.

## Conclusion

This work deals with a polyptych painted by Pietro Lorenzetti in 1320. Two mock-up samples and the corresponding geometric models were made based on that polyptych. By using the geometric model of the sample, numerical simulation was established to simulate the experimental process and results. After numerical simulation, two samples were tested in a real experimental environment, and the actual surface temperature images of the two samples were collected. In order to identify the defects in the two samples, a LRTDTV denoising system was proposed, so as to reduce the noise and enhance the contrast of the infrared thermal images. Quantitative analysis was conducted to verify the performance of the proposed algorithm.

Some encouraging outcomes are found. First, it is found that the surface temperature obtained via numerical simulation has a good match to the experimental one. Secondly, the Gaussian noise in thermographic images can be effectively eliminated by the LRTDTV model. Through the observation of the experimental results after treatment, it is found that the defects in the samples can be detected easily by IRT without damaging the sample. Finally, through this approach, the difference between the thermal conductivity and the heat capacity at constant pressure of different materials can be used to detect the buried and unknown defects in artworks.

## Data Availability

Data and material are available at reasonable request.
